# Faster smooth muscle cell coverage in ultrathin-strut drug-eluting stent leads to earlier re-endothelialization

**DOI:** 10.3389/fbioe.2023.1207858

**Published:** 2023-05-23

**Authors:** Dongwoo Hahn, Donghoon Lee, Woonggyu Hyun, Yunnie Cho, Chang-Hwan Yoon, Ki-Hyun Jeon, Si-Hyuck Kang, Tae-Jin Youn, In-Ho Chae

**Affiliations:** ^1^ Division of Cardiology, Department of Internal Medicine, Seoul National University Bundang Hospital, Seongnam, Republic of Korea; ^2^ Cardiovascular center, Asan Chungmu Hospital, Asan, Republic of Korea; ^3^ College of Medicine, Seoul National University, Seoul, Republic of Korea

**Keywords:** drug-eluting stent (DES), optical coherence tomography, re-endothelialization, neointimal coverage, smooth muscle cell (SMC)

## Abstract

**Background:** The ultrathin-strut drug-eluting stent (DES) has shown better clinical results than thin- or thick-strut DES. We investigated if re-endothelialization was different among three types of DES: ultrathin-strut abluminal polymer-coated sirolimus-eluting stent (SES), thin-strut circumferential polymer-coated everolimus-eluting stent (EES), and thick-strut polymer-free biolimus-eluting stent (BES) to gain insight into the effect of stent design on promoting vascular healing.

**Methods:** After implanting three types of DES in the coronary arteries of minipigs, we performed optical coherence tomography (OCT) at weeks 2, 4, and 12 (*n* = 4, each). Afterward, we harvested the coronary arteries and performed immunofluorescence for endothelial cells (ECs), smooth muscle cells (SMCs), and nuclei. We obtained 3D stack images of the vessel wall and reconstructed the en face view of the inner lumen. We compared re-endothelialization and associated factors among the different types of stents at different time points.

**Results:** SES showed significantly faster and denser re-endothelialization than EES and BES at weeks 2 and 12. Especially in week 2, SES elicited the fastest SMC coverage and greater neointimal cross-sectional area (CSA) compared to EES and BES. A strong correlation between re-endothelialization and SMC coverage was observed in week 2. However, the three stents did not show any difference at weeks 4 and 12 in SMC coverage and neointimal CSA. At weeks 2 and 4, SMC layer morphology showed a significant difference between stents. A sparse SMC layer was associated with denser re-endothelialization and was significantly higher in SES. Unlike the sparse SMC layer, the dense SMC layer did not promote re-endothelialization during the study period.

**Conclusion:** Re-endothelialization after stent implantation was related to SMC coverage and SMC layer differentiation, which were faster in SES. Further investigation is needed to characterize the differences among the SMCs and explore methods for increasing the sparse SMC layer in order to improve stent design and enhance safety and efficacy.

## Highlights

· Neointimal coverage observed by optical coherence tomography and histologic coverage of vascular smooth muscle cells were almost complete after four weeks.
· However, histological re-endothelialization reached 50% at four weeks and 75% at 12 weeks after stent implantation.
· Re-endothelialization of stent struts at the late phase was associated with underlying neointimal smooth muscle cell phenotype.

## Introduction

Since the introduction of angioplasty using balloons in 1977, bare-metal stents (BMS) in the 1980s, and drug-eluting stents (DES) in the 2000s, vascular healing of an injured coronary artery involving neointimal hyperplasia and re-endothelialization has been a major target to improve clinical efficacy after percutaneous coronary intervention (PCI) (Stefanini and Holmes, 2013; Fischman et al., 1994; Nicolas et al., 2022). In the process of PCI, endothelial cells (ECs) of blood vessels are inevitably damaged, and smooth muscle cells (SMCs) are activated in the subsequent recovery process, resulting in hyper-proliferation of intima tissue (Stefanini et al., 2017). Although antiproliferative agents that inhibit SMC proliferation significantly reduce restenosis, the risk of late stent thrombosis (LST) via suppression of endothelial regrowth remains a major concern after PCI, even with contemporary DES, such as thin-strut everolimus-eluting stents (EES), thick-strut polymer-free biolimus-eluting stents (BES), and ultra-thin strut biodegradable-polymer sirolimus-eluting stents (SES) (Taniwaki et al., 2016).

Intracoronary imaging, including intravascular ultrasound (IVUS) and optical coherence tomography (OCT), is used to clinically estimate the re-endothelialization of the strut surface and predict the risk of LST. OCT is the method of choice to characterize fibrous tissue, plaque, thrombus, and neointimal coverage, due to its axial resolution of 5–20 μm. Recently, it has been reported that the evaluation of strut coverage by OCT can be one of the factors for clinicians to decide the duration of dual antiplatelet therapy (Iliescu et al., 2017; Lee et al., 2018). However, it has been reported that neointimal coverage, which can be monitored by OCT, does not always include re-endothelialization of the vessel wall and fails to reveal the mechanism of clinical outcome difference among the stents. This grants closer histological observation after stent implantation over time (Torii et al., 2020). Therefore, it is necessary to investigate why contemporary stents show different clinical outcomes, particularly in terms of re-endothelialization, which is the true endpoint of vascular healing after stent implantation.

In this preclinical study, we investigated the relation between re-endothelialization, SMC pathophysiology, and neointimal coverage after stent implantation using quantitative histological examination and OCT data. Particularly, we compared the process of SMC differentiation and its effect on re-endothelialization after the implantation of EES, BES, and SES in the coronary artery of a porcine model.

## Materials and methods

### Animal care and use

The animal experimental procedures were approved by the Institutional Animal Care and Use Committee (IACUC) of Seoul National University Bundang Hospital (BA 1808-253/067-03) and performed in accordance with the Guide for the Care and Use of Laboratory Animals from the Institute of Laboratory Animals Resources. The day before the experiment, male minipigs (*n* = 12; body weight = 25–35 kg) were administered aspirin (300 mg) and clopidogrel (300 mg). On the day of the experiment, the pigs were premedicated with atropine sulfate (0.05 mg/kg, intramuscularly) and subsequently anesthetized with zoletil (5 mg/kg, intramuscularly) and xylazine (4.4 mg/kg, intramuscularly). Afterward, the pigs were intubated and ventilated with room air and sevoflurane. We inserted a 6 Fr sheath via the right femoral artery using an ultrasound-guided puncture.

### Design

This study was conducted using three different stents: EES (Xience Sierra™, Abbott Vascular, United States), BES (Biofreedom™, Biosensors, United States), and SES (Genoss DES™, GENOSS, South Korea). The thin-strut EES has 81-μm-thick cobalt–chromium struts and a PVDF-HFP polymer-containing everolimus (Table 1). The thick-strut BES has 119-μm-thick stainless steel struts and releases biolimus A9 without a polymer. The ultrathin-strut SES has 70-μm-thick cobalt–chromium struts and a PLLA/PLGA biodegradable polymer-containing sirolimus.

**TABLE 1:** Characteristics of contemporary coronary drug-eluting stents tested in this study.

Stent	Drug	Stent material	Strut thickness (μm)	Polymer	Polymer type	Coating distribution	Polymer thickness
BES (Biofreedom™)	Biolimus A9	316 L	119	Polymer free	Polymer-free	Abluminal	Polymer-free
		Stainless steel					
EES (Xience Sierra™)	Everolimus	L-605	81	PVDF-HFP	Durable	Conformal	7–8 μm
		CoCr					
SES (Genoss-DES^TM^)	Sirolimus	L-605	70	PLLA/PGLA	Bioresorbable	Abluminal	<3 μm
		Co-Cr					

CoCr, cobalt–chromium; PGLA, poly(lactic-co-glycolic acid); PLLA, poly(L-lactic acid); PVDF-HFP, copolymer of vinylidene fluoride and hexafluoropropylene.

**FIGURE 1:**
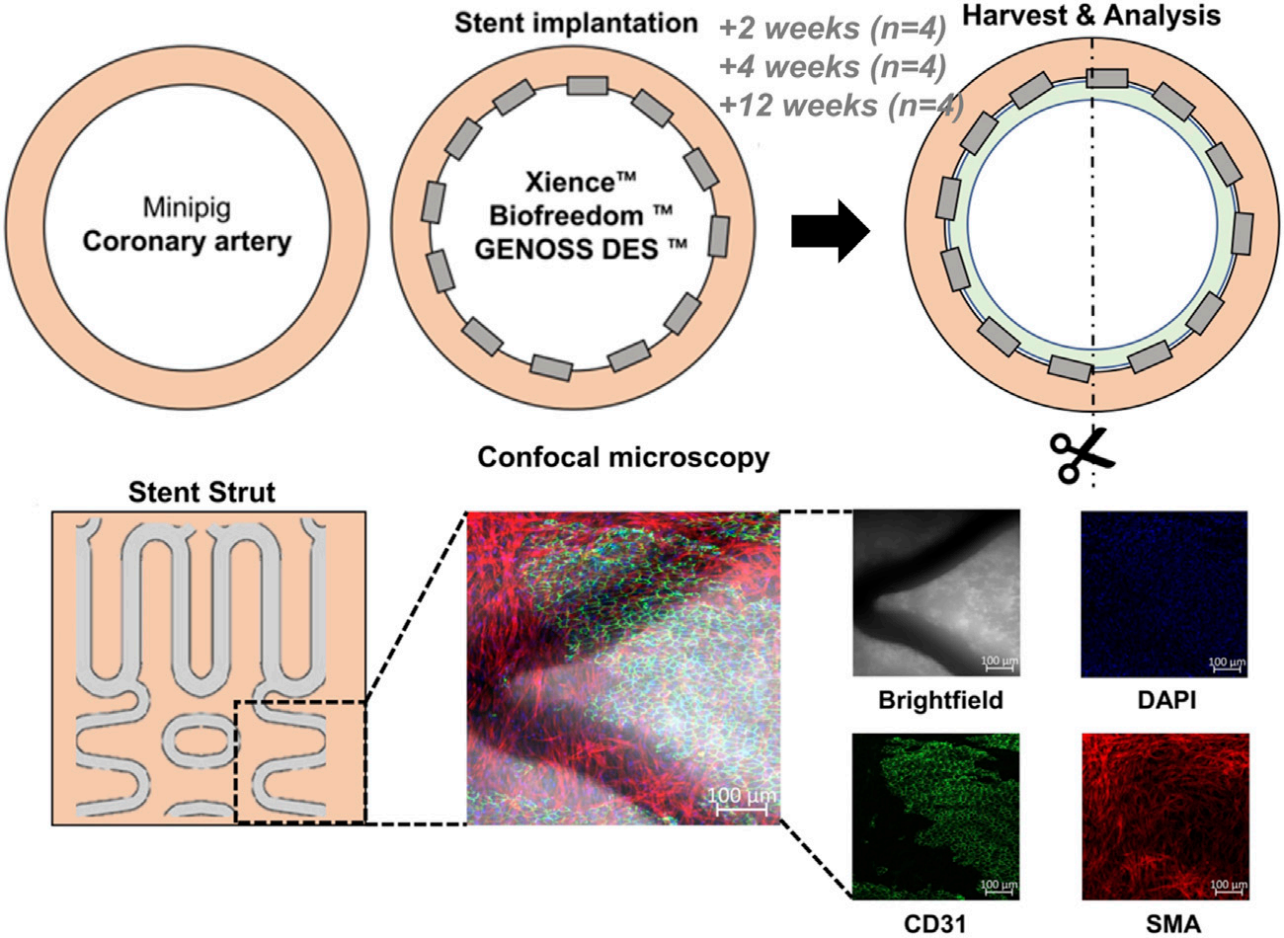
General schema of the experiment. Three different drug-eluting stents were implanted into the three coronary arteries (the left anterior descending artery, left circumflex artery, and right coronary artery) of a minipig. The arteries were harvested at 2, 4, and 12 weeks after stent implantation. Cells were analyzed under a confocal microscope after immunofluorescence staining with DAPI for nuclei, CD31 for endothelial cells, and smooth muscle actin (SMA) for smooth muscle cells. BES, Biofreedom^TM^; EES, Xience Sierra^TM^; SES, Genoss-DES^TM^.

These three stents were implanted in the three coronary arteries of 12 pigs. After stent implantation, the pigs were dosed with 100 mg aspirin and 75 mg clopidogrel daily. We assigned each of the four pigs to 2-, 4-, and 12-week post-stent implantation groups and compared the results from OCT and histology (Figure 1).

### OCT imaging

Coronary angiography and OCT were performed using a Dragonfly^®^ image catheter and C7-XR™ OCT system (Abbott, United States) after an intra-coronary injection of nitroglycerine before autopsy. From the OCT images, the cross-sectional area (CSA) of the coronary artery and stent strut apposition or coverage were evaluated using a dedicated imaging analysis program (OPTIS™ Imaging Systems software, Abbott Korea, Seoul, Korea).

### Scanning electron microscopy

Samples were fixed in 10% neutral buffered formalin at room temperature. Dehydration treatment was performed using 50%–100% ethanol. Blood vessels were longitudinally cut, opened to expose the endothelium, and dried naturally. All samples were coated with gold to impart conductivity. The samples were imaged after selecting the magnification while appropriately adjusting the electron beam voltage and working distance by field-emission scanning electron microscopy (Sigma 500, Zeiss, Germany).

### Histologic analysis

After the coronary arteries were harvested, endothelialization and neointima growth were evaluated by staining with antibodies against CD31, a glycoprotein expressed on ECs, alpha-smooth muscle actin (SMA-cy3), and nucleic acid (DAPI, 4,6-diamidino-2-phenylindole). Histological quantification was performed by obtaining three to six random field images from each coronary artery (Figure 1). Confocal microscopy was performed using a Zeiss LSM800 microscope (Carl Zeiss Korea, Seoul, Korea). Maximal intensity projection images were reconstructed from 100-fold magnification Z-stacked images. The total area of the stent strut and the area of the stent strut covered by ECs or SMCs were identified by immunofluorescence staining and quantified using the ImageJ program (Java-based image processing program, NIH, version 1.48s).

### Statistical analysis

Continuous variables are reported as the mean ± standard deviation. One-way ANOVA and a statistical model constructed by generalized estimating equations with a variable number of repeated measures on every millimeter of OCT images and random field histologic images of each stent were applied. The main independent variable was the type of stent implanted, whereas the covariates included the major branches of the coronary artery where the stent was implanted. The dependent variables included the CSA of the stent, lumen, and neointima on OCT images, as well as the area of the stent strut, the area of the stent strut covered by ECs, or SMCs on histologic images. If there was significance among the three groups, Bonferroni’s *post hoc* test was performed to find the statistical significance of the difference between one group and another. Statistical analyses were performed using R (R Foundation for Statistical Computing, Vienna, Austria; http://www.R-project.org, version 3.6.1). A two-sided *p*-value <0.05 was considered statistically significant.

## Results

### Time-dependent vascular healing

**FIGURE 2:**
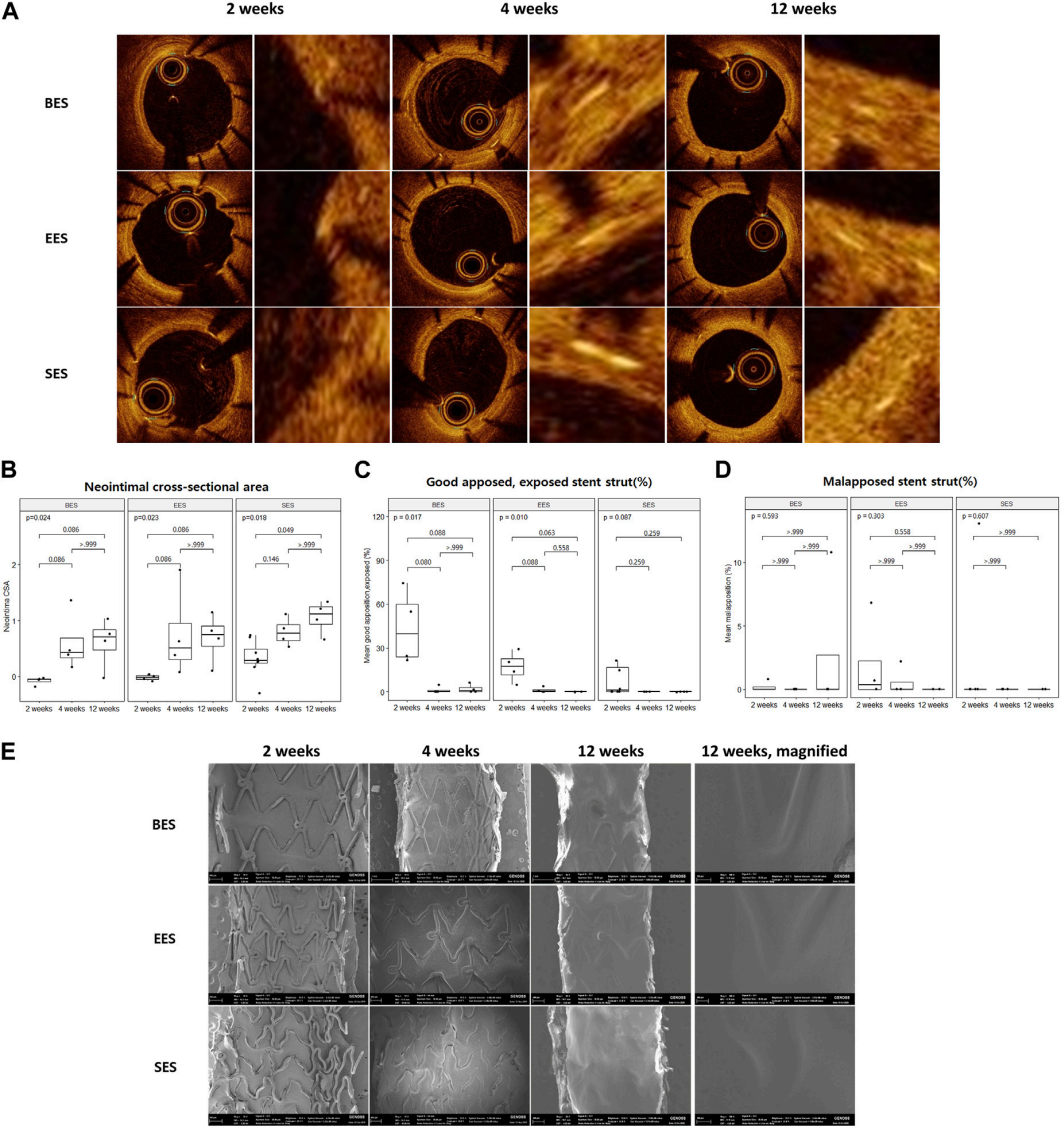
Neointimal coverage after stent implantation by optical coherence tomography and scanning electron microscopy. **(A)** Representative image of sequential optical coherence tomography images of stented coronary artery lumen at 2, 4, and 12 weeks after stent implantation. Images on the right pair show a magnified view of the neointimal area of the vessel wall. **(B)** Neointimal cross-sectional area, **(C)** % good apposition, exposed strut, and **(D)** % malapposed strut at three-time points. **(E)** Representative image of sequential scanning electron microscopic images of the stented vessel lumen at 2, 4, and 12 weeks after stent implantation. BES, Biofreedom™; EES, Xience Sierra™; SES, Genoss-DES^TM^.

All pigs survived the in-life phase of the study. All stents were evenly expanded at a 1:1 stent-to-vessel ratio. Angiography and OCT were performed immediately after stent implantation and at 2, 4, and 12 weeks (Figure 2A). No significant in-stent restenosis or stent thrombosis was observed by angiography or OCT throughout the study. CSA of the neointima gradually increased over time in all groups (Figure 2B). At week 2, the neointimal CSA in the SES group was significantly greater than in the BES and EES groups (Table 2). However, neointimal CSA showed no difference among the three stent groups at weeks 4 and 12 (Table 2). In terms of stent coverage, there was no statistical difference in the percentage of exposed and malapposed struts during the experimental period (Figures 2C, D; Table 2). Scanning electron microscopy also showed rapid neointimal growth and nearly complete stent coverage at week 12 after implantation in all stent groups (Figure 2E).

**TABLE 2:** Optical coherence tomography analysis for each stent at 2, 4, and 12 weeks after implantations.

Time	Variable	BES	EES	SES	F-test *p*-value
2 weeks	Neointima CSA (mm^2^)	−0.08 ± 0.07	−0.02 ± 0.05	0.32 ± 0.78	0.028^a^
	% Good apposed, covered	55.9 ± 25.4	81.0 ± 9.8	82.8 ± 34.1	0.303
	% Good apposed, exposed	43.9 ± 25.2	17.1 ± 10.3	15.6 ± 29.7	0.202
	% Malapposition	0.2 ± 0.4	1.9 ± 3.3	1.6 ± 4.6	0.781
4 weeks	Neointima CSA (mm^2^)	0.60 ± 0.52	0.75 ± 0.80	0.80 ± 0.25	0.878
	% Good apposed, covered	98.8 ± 2.3	98.3 ± 3.0	100.0 ± 0.0	0.557
	% Good apposed, exposed	1.2 ± 2.3	1.2 ± 1.9	0.0 ± 0.0	0.576
	% Malapposition	0.0 ± 0.0	0.6 ± 1.1	0.0 ± 0.0	0.405
12 weeks	Neointima CSA (mm^2^)	0.61 ± 0.45	0.69 ± 0.43	1.06 ± 0.29	0.279
	% Good apposed, covered	95.3 ± 6.0	10.0 ± 0.0	100.0 ± 0.0	0.143
	% Good apposed, exposed	2.0 ± 3.0	0.0 ± 0.0	0.0 ± 0.0	0.217
	% Malapposition	2.7 ± 5.4	0.0 ± 0.0	0.0 ± 0.0	0.405

BES, Biofreedom™; EES, Xience Sierra™; SES, Genoss-DES^TM^; CSA, cross-sectional area; Mean ± SD.

^a^ One-way ANOVA, with Bonferroni’s *post hoc* test; BES vs. SES *p* = 0.054; BES vs. EES *p* > 0.999; SES vs. EES *p* = 0.115.

**FIGURE 3:**
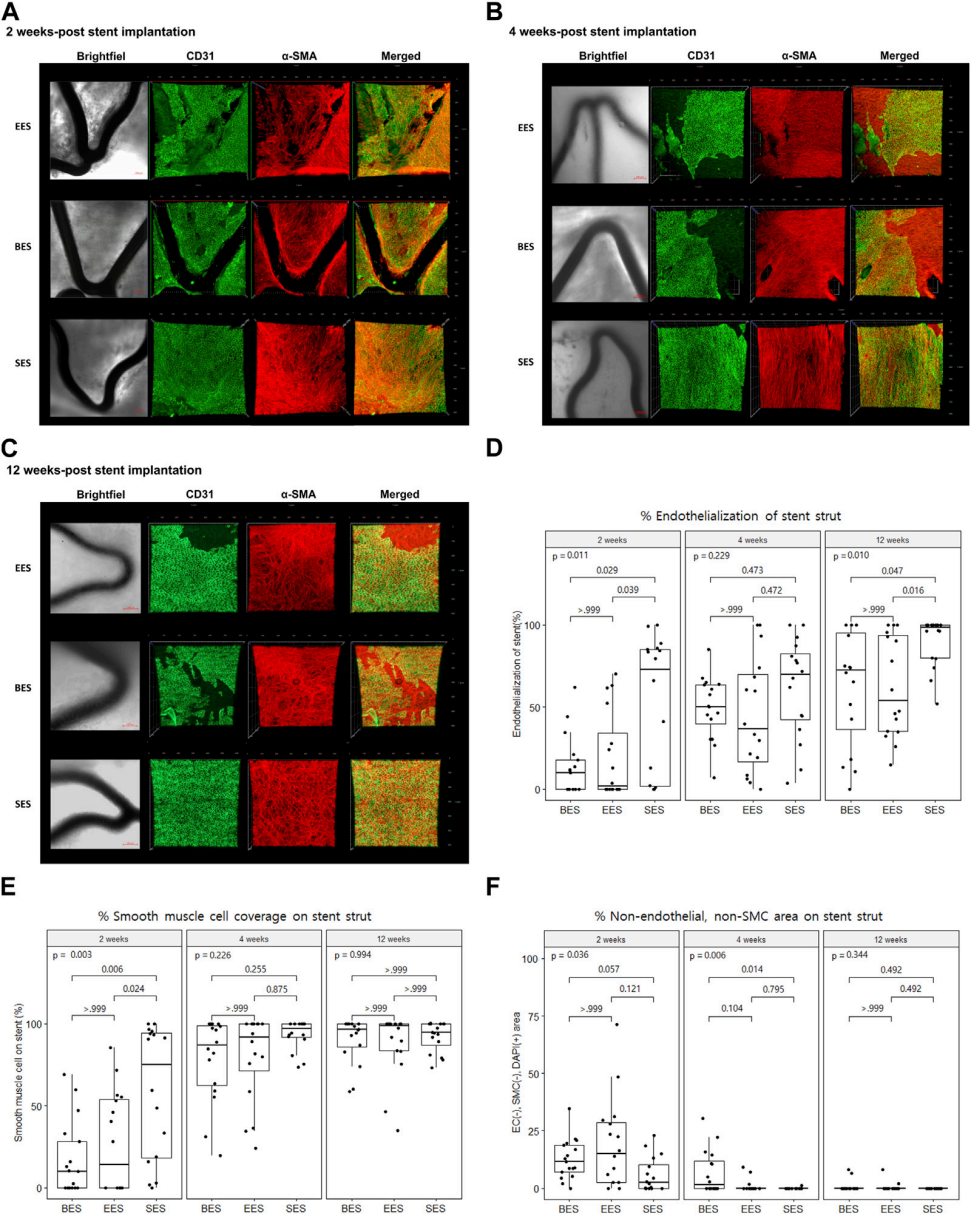
Re-endothelization and smooth muscle cell coverage after stent implantation by immunofluorescence. **(A–C)** Representative immunofluorescence images of stented coronary artery tissue depicting the endothelium (CD31, green), smooth muscle cell (α-smooth muscle actin, red), merged, and brightfield at 2, 4, and 12 weeks after stent implantation. Brightfield images show the stent strut (dark area). Scale bar = 100 μm. **(D)** % area of endothelial cells on different stent struts at 2, 4, and 12 weeks after stent implantation. **(E)** % area of smooth muscle cells on different stent struts at 2, 4, and 12 weeks after stent implantation. **(F)** % area of non-endothelial, non-smooth muscle cells on different stent struts at 2, 4, and 12 weeks after stent implantation. SMC, smooth muscle cell; BES, Biofreedom™; EES, Xience Sierra™; SES, Genoss-DES^TM^.

Using confocal microscopic images, we identified stent strut endothelialization and SMC coverage, as shown in Figures 3A–C. We found that endothelialization was slow and was not finished until week 12, whereas SMC coverage was fast and almost complete within 4 weeks (Figures 3D, E). Evaluation of cellularity using the DAPI (+) area on the strut showed that cellularity gradually decreased during the study period. Particularly, non-endothelial and non-SMCs, which were abundant at week 2, dramatically decreased at week 12 (Figure 3F).

The proportion of the area covered with ECs in the SES group was statistically greater than that in the BES and EES groups at week 2 (SES: 51.8 ± 41.6; BES: 13.3% ± 18.1%; EES: 19.7% ± 26.7%) (Figures 3A, D). At week 4, there was no significant difference in the proportion of the area covered with ECs among the three groups (Figures 3B, D). However, at week 12, the endothelial coverage area in the SES group (90.0% ± 15.1%) was significantly greater than that in the BES group (61.8% ± 35.2%, *p* = 0.100) or EES (62.2% ± 30.7%) (Figures 3C, D). The SMC coverage of the SES group progressed faster than that in BES and EES groups at week 2, but there was no difference in weeks 4 and 12 (Figure 3E). The area where only the nuclei were stained without endothelial or SMC marker staining was significantly smaller in the SES group compared to the BES group at week 4 (Figure 3F).

### Delayed re-endothelialization compared to neointimal or SMC coverage

**FIGURE 4:**
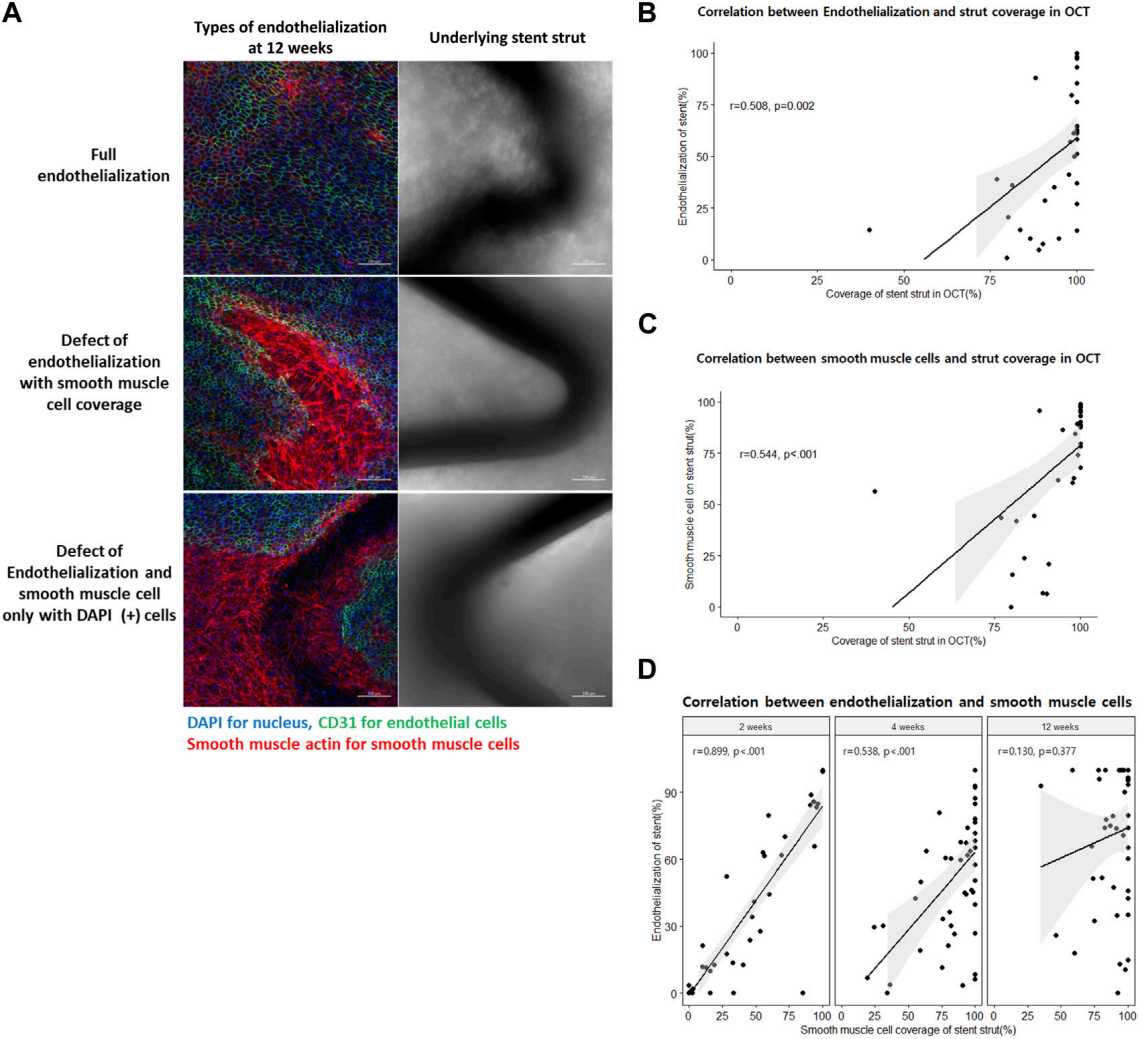
Delayed re-endothelialization and its predictors. **(A)** Different types of endothelialization at 12 weeks after stent implantation on the luminal side of the arterial wall by immunofluorescence. Scale bar = 100 μm (blue = nucleus, green = endothelial cells, and red = smooth muscle cells). **(B)** Correlation between the histologic endothelialization on the stent strut and optical coherence tomography (OCT)-defined covered stent strut. **(C)** Correlation between the histologic smooth muscle cell (SMC) coverage on the stent strut and OCT-defined covered stent strut. **(D)** Correlation between the histologic endothelialization and SMC coverage on the stent struts at 2, 4, and 12 weeks after stent implantations. OCT, optical coherence tomography; SMC, smooth muscle cells.

We observed full re-endothelialization on the neointima 12 weeks after stent implantation, and we found vessel wall defects of endothelium on neointima or even uncovered stent struts (Figure 4A). We evaluated the correlation between stent strut coverage from OCT analysis versus endothelialization and versus SMC coverage from histology. There was a correlation, but a gap between OCT and histologic quantification was observed. Specifically, 100% stent coverage observed by OCT was matched to around 55% in endothelialization and 75% in smooth muscle cells on the stent strut by histology (Figures 4B, C). Furthermore, there was a significant correlation between SMC coverage and re-endothelialization of stent struts at week 2 (Pearson’s *r* = 0.899, *p* < 0.001). However, there was a very weak correlation at week 4 (Pearson’s *r* = 0.538, *p* < 0.001) and no statistical significance at week 12 (Pearson’s *r* = 0.130, *p* = 0.377) (Figure 4D).

### Effect of smooth muscle cell morphology on re-endothelialization

**FIGURE 5:**
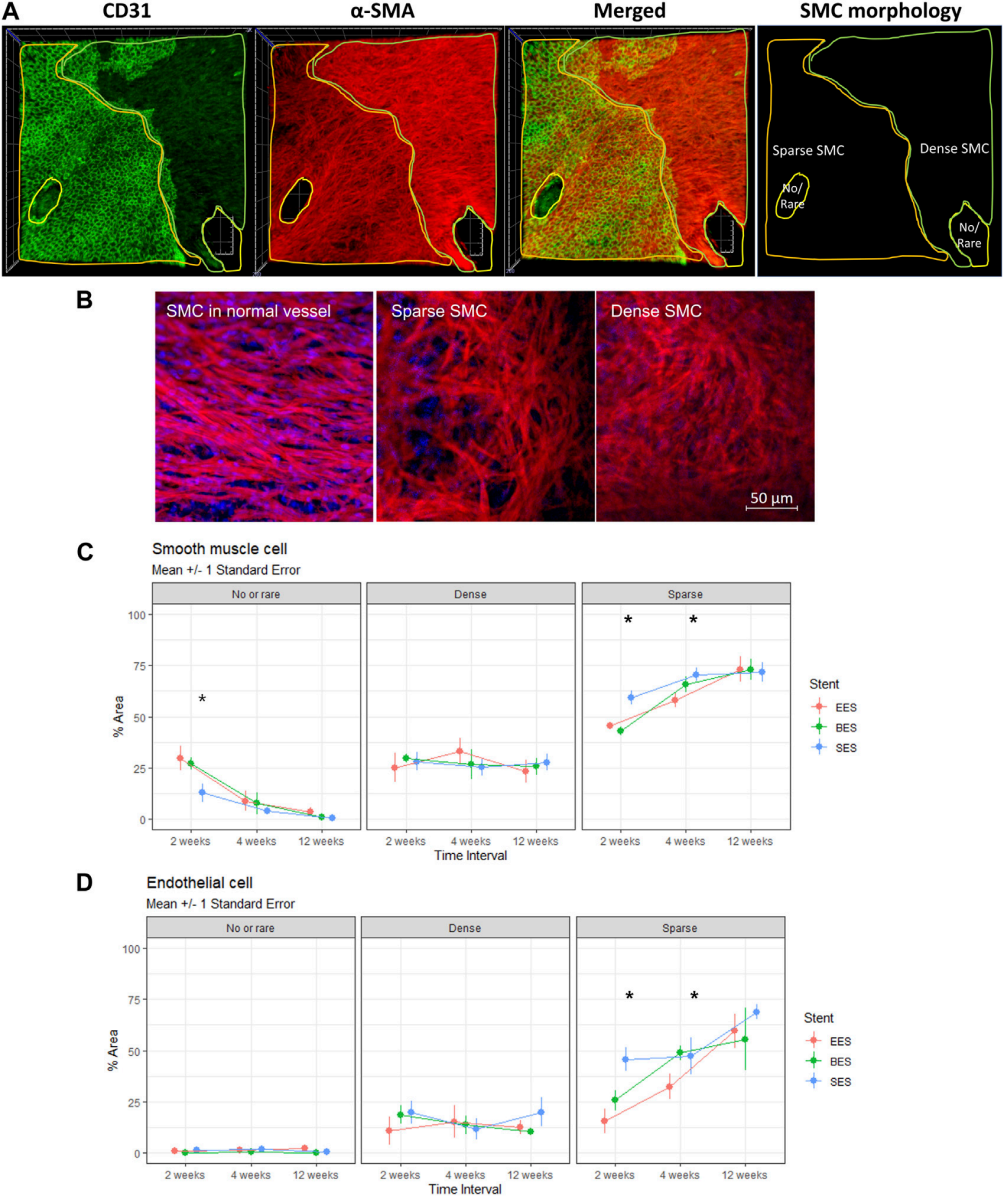
Morphology of smooth muscle cells and its effect on re-endothelialization. **(A)** Representative immunofluorescence image quantification of stented coronary artery tissue showing no/rare, dense (spindle), or sparse (stellate) SMC distribution on the luminal side of the stented coronary artery at 4 weeks after stent implantation. Endothelium (CD31, green), SMC (α-smooth muscle actin, red), merged, and SMC morphology distribution. Side of the rectangle = 800 μm. **(B)** Representative morphologic difference of SMC. Left panel, SMC in the normal vessel; mid panel, sparse SMC; right panel, dense SMC. **(C)** % area of no/rare, dense, or sparse SMC at 2, 4, and 12 weeks after stent implantations. *p < 0.05. **(D)** % area of endothelial cell distribution in the regions of no/rare, dense (spindle), or sparse (stellate) SMC distribution at 2, 4, and 12 weeks after stent implantation. *p < 0.05. BES, Biofreedom™ in green; EES, Xience Sierra™ in red; SES, Genoss-DES^TM^ in blue; SMC, smooth muscle cells.

We observed SMCs of different morphologies on the luminal side of the coronary arteries: no/rare, sparse (stellate), and dense (spindle) (Figure 5A). SMCs in normal vessels showed parallel alignment and vivid interconnecting muscle fibers (Figure 5B). However, sparse SMCs lost alignment but had connecting fibers. In contrast, dense SMCs showed high cellularity and poor development of interconnecting fibers. Throughout the experiment, the no/rare SMC area was decreased, whereas sparse SMC was increased (Figure 5C). However, the dense SMC area did not change. The no/rare SMC area was significantly lower in the SES group compared to the EES and BES groups at week 2 (*p* = 0.005). The sparse SMC area was significantly different at weeks 2 and 4, higher in the SES group (*p* < 0.001 and *p* = 0.010, respectively). Interestingly, the re-endothelialization on each SMC morphology layer was significantly different. On the no/rare SMC area, approximately 1% of the area showed re-endothelialization (Figure 5D; Table 3). Similarly, 13.5%–16.5% of the area showed re-endothelialization in the dense SMC area. On the contrary, a significantly greater re-endothelialization area of 29.0%–61.3% was found in the sparse SMC area. Re-endothelialization increased as time passed only in the sparse SMC area. Particularly, re-endothelialization in the sparse SMC area was significantly higher in SES at weeks 2 and 4 (Figure 5D).

**TABLE 3:** Re-endothelialization rate by smooth muscle cell morphology underneath.

Time	Smooth muscle cell morphology	Re-endothelialization (%)	*p*-value by generalized estimating equation	
			Within	Between
2 weeks	No/rare	1.0 ± 3.2	Reference	<0.001*
	Dense	16.5 ± 14.6	<0.001*	
	Sparse	29.0 ± 23.1	<0.001*	
4 weeks	No/rare	1.3 ± 3.2	Reference	<0.001*
	Dense	13.5 ± 22.1	<0.001*	
	Sparse	42.8 ± 23.3	<0.001*	
12 weeks	No/rare	1.0 ± 3.4	Reference	<0.001*
	Dense	14.3 ± 17.4	<0.001*	
	Sparse	61.3 ± 26.0	<0.001*	

Mean ± SD. *p < 0.05 by generalization estimating equations with Bonferroni’s *post hoc* test.

## Discussion

This study demonstrates the difference between neointimal coverage and re-endothelialization of stents in the coronary artery and the effect of stent design on endothelialization after different types of DES implantation. Neointimal coverage of the stent struts was almost complete at 4 weeks after implantation, but re-endothelialization was not completed until week 12. Re-endothelialization seemed to be affected by the underlying SMC layer, particularly in the early phase of vascular healing, as there was a correlation between the appearance of the endothelium and the stent coverage of SMC until 4 weeks. In addition, we found no significant difference in the neointimal coverage of each type of stent tested in this study observed by the OCT. However, there was a significant difference in the endothelial coverage of the struts of each stent. The ultra-thin strut and abluminal biodegradable polymer SES showed the best re-endothelialization in the early and late phases of vascular healing. Re-endothelialization in the late phase of vascular healing seemed to be affected by the underlying SMC morphology as re-endothelialization progressed only on the sparse SMC area.

Stent thrombosis is caused by delayed healing of the coronary artery. It is also affected by persistent fibrin deposition, hypersensitivity, bifurcation lesions, and stent-strut malapposition (Joner et al., 2006). Especially after stent implantation, delayed re-endothelialization is an important factor in stent thrombosis (Finn et al., 2007; Otsuka et al., 2014). Finn et al. (2007) stated that endothelial coverage of the stent strut is the most powerful histological predictor of stent thrombosis, and the ratio of uncovered stent strut is the best morphometric predictor of stent thrombosis. Early stent thrombosis (within 1 month) is primarily affected by interventional factors, such as under-expansion, stent edge dissection, plaque rupture, and medial fracture. However, most cases of LST (1–12 months) are affected by stent strut exposure due to delayed arterial healing (Nakazawa et al., 2011).

Imaging methods, such as IVUS or OCT, are used to clinically evaluate re-endothelialization of the stent strut and assess the risk of LST indirectly as it is impossible to evaluate re-endothelialization of the coronary arteries histologically. OCT offers the highest resolution among all the currently available coronary imaging modalities (axial 10–20 μm and lateral 20–90 μm), which is ∼10 times greater than that of IVUS (Taniwaki et al., 2016). Furthermore, OCT is an optimal approach to characterize fibrous tissue, plaque, thrombus, and neointimal coverage in the context of vascular healing. According to Lee et al. (2018), strut coverage can be evaluated by OCT, and the prescription of dual antiplatelet therapy (DAPT) for 3 months can be considered based on the results. Iliescu et al. (2017) argued that risk factors for stent thrombosis, such as stent malapposition and uncovered stent strut, can be evaluated using OCT, and DAPT can be discontinued based on the results. However, other studies showed that more stent thrombosis was found in the group with more advanced neointimal growth detected by OCT, suggesting that strut coverage findings in OCT and IVUS reflect just neointimal coverage, which does not imply complete endothelial cell coverage (Murakami et al., 2009). In the present study, we found that strut coverage evaluated by OCT should not be interpreted as complete re-endothelialization, which was far more delayed than neointimal coverage. Therefore, it can be dangerous to assess the risk of stent thrombosis based on strut coverage observed by OCT. As the period of DAPT after PCI should be determined based on the risk of bleeding and ischemic events, the optimal period of DAPT remains controversial. According to SMART CHOICE (Hahn et al., 2019) and STOPDAPT-2 trials (Watanabe et al., 2019), the efficacy of short DAPT was not inferior to longer DAPT in terms of cardiovascular and cerebrovascular outcomes. These studies suggest that single antiplatelet therapy can be an option to prevent thrombotic events despite incomplete re-endothelialization, as shown in the present study. However, the SMART-DATE trial showed that the risk of major adverse cardiac and cerebrovascular events in patients with acute coronary syndrome seemed to be higher in the 6-month DAPT group than in the 12-month or longer DAPT group (Hahn et al., 2018). Re-endothelialization in patients with acute coronary syndrome may be delayed, and thrombotic events may occur after the cessation of DAPT. Therefore, re-endothelialization in certain pathological conditions may show more variability. Further study is required to investigate the complex process of re-endothelialization.

One possible indication for re-endothelialization is SMC. There was a correlation between stent coverage of the SMC and re-endothelialization until 4 weeks after implantation. SMC coverage was much faster than re-endothelialization. Therefore, re-endothelialization seemed to require an underlying SMC layer, particularly in the early phase of vascular healing. In addition, the shape of the SMC on the surface of the stent strut changed from dense and spindle to sparse and stellate over time in the present study. SMC is known to change its shape and respond to damage once vascular damage occurs. Specifically, spindle-shaped SMCs are known to transform into a stellate shape by various factors, including miRNA, to promote the recovery of blood vessels (Davis-Dusenbery et al., 2011). The contractile phenotype of spindle shape is characterized by low proliferation. In contrast, the synthetic phenotype of stellate shape is characterized by increased proliferation, migration, and extracellular matrix production (Liu and Gomez, 2019). In the present study, the changes in SMC occurred gradually over 4 weeks and up to 12 weeks after stent implantation. When data for the entire period were analyzed, the sparse SMC layer showed a significantly higher rate of re-endothelialization than the dense SMC layer.

Stent design influences re-endothelialization and intimal hyperplasia (Soucy et al., 2010). Blood flow disturbance and shearing stress caused by the implanted stent affect endothelial growth (Hsiao et al., 2016). Analysis of endothelial cell recovery between DES showed that the thinner stent strut/polymer leads to more endothelial cell coverage. Stent design characteristics can affect thromboresistance, speed of neointimal coverage, and re-endothelialization, thus influencing the duration of DAPT after coronary intervention (Torii et al., 2020). The second-generation DES was shown to have less inflammation, fibrin deposition, and stent thrombosis than the first-generation DES with improved strut coverage (Otsuka et al., 2014). However, the antiproliferative agent used in DES inhibits the proliferation of vascular ECs and SMCs. In particular, mTOR inhibitors, also used in the present study, are known to inhibit the growth and repair of ECs by inhibiting the mTORC1 complex by binding to FKBP12, which causes incomplete re-endothelialization of stent struts (Habib and Finn, 2015). In the present study, SES showed the best initial neointimal growth on OCT and re-endothelialization at 2 weeks after implantation. The ultrathin-strut and abluminal polymer might cause SMCs to proliferate and migrate faster on the stent strut, resulting in higher SMC coverage and re-endothelialization than the others. In addition, SES showed a significantly higher sparse SMC layer at weeks 2 and 4, which seemed to result in a higher re-endothelialization at weeks 2 and 12 in the present study. In line with this preclinical observation, a recent prospective registry of SES showed excellent clinical results (Youn et al., 2020). In contrast, the vascular healing represented by endothelialization by polymer-free BES was inferior to SES and similar to EES. BES was expected to improve vascular healing, as shown in a previous clinical study in patients with a high bleeding risk (Urban et al., 2015). However, the strut thickness of the BES might hinder the initial SMC and endothelial migration on the stent strut, finally leading to delayed re-endothelialization in the present study.

### Limitations

This study had several limitations. First, the healing processes in humans and pigs are not the same. Neointimal responses are pronounced, and the time course of healing is five-to-six times longer in humans than in animals (Virmani et al., 2003). This study examined the re-endothelialization process up to 12 weeks after stent implantation in a porcine model, which may be equivalent to the healing process in humans beyond 1 year. However, our model is anatomically and physiologically similar to humans compared to other animals (Nakazawa et al., 2007). Second, we observed the process of re-endothelialization after a stent was implanted into a normal blood vessel without atherosclerosis. Therefore, the implantation of a DES into blood vessels with atherosclerosis or thrombus may differ from the observations in this study. Third, sirolimus, everolimus, and biolimus share the key mechanism to inhibit SMC growth as mTOR inhibitors. However, the three different kinds of mTOR inhibitors can have different effects on endothelialization independent of stent design.

## Conclusion

Re-endothelialization after stent implantation was related to SMC coverage at the early phase and SMC layer differentiation at the late phase. Ultrathin-strut SES showed faster recovery than thin-strut EES or thick-strut BES. The pathophysiology of SMC and its characterization and modification should be further investigated to improve stent design to improve re-endothelialization and its clinical efficacy and safety.

### Clinical perspectives

#### Clinical competencies

OCT is frequently used to clinically estimate re-endothelialization of stent struts, indirectly assessing the risk of stent thrombosis as it is impossible to test re-endothelialization of the coronary arteries histologically. In this study, we found that it is not the neointimal coverage observed on OCT that is critical, but that the SMC layer and its differentiation is essential for the re-endothelialization of the stented vascular wall. Therefore, the pathophysiology of SMC and its modification should be further investigated to improve stent design to improve re-endothelialization and its clinical efficacy and safety.

#### Translational outlook

In this study, the DES design affects the rapidity of SMC coverage and morphology of the SMC layer, leading to different re-endothelialization and vascular healing. Therefore, it is necessary to further renovate the coronary stent to enhance the initial proliferation and differentiation of SMC, which can improve re-endothelialization.

## Data Availability

The original contributions presented in the study are included in the article/supplementary material. Further inquiries can be directed to the corresponding author.
